# Development and application of a TaqMan-MGB probe-based quantitative real-time polymerase chain reaction assay for the rapid detection of *Dictyocaulus filaria*

**DOI:** 10.3389/fvets.2025.1559088

**Published:** 2025-08-11

**Authors:** Zheng-qin Gao, Jin Xing

**Affiliations:** National Institutes for Food and Drug Control (NIFDC), Beijing, China

**Keywords:** lungworm disease, *Dictyocaulus filaria*, TaqMan-MGB-probe, LLOQ, LOD

## Abstract

Lungworm disease caused by *Dictyocaulus filaria* is an infectious condition affecting sheep and goats worldwide in recent years. It causes significant economic losses and is considered a potential public health threat. To date, there have been few studies on *Dictyocaulus filaria*. Its pathogenic mechanism is still unclear, and there are no effective vaccines or drugs available for the prevention and control of the disease. Therefore, it is essential to develop a rapid and reliable molecular diagnostic method to facilitate the study of this novel parasite. In this study, we developed a TaqMan-minor groove binder (MGB) probe-based quantitative real-time polymerase chain reaction (qPCR) for the rapid detection of *Dictyocaulus filaria* for the first time. Specific primers and a TaqMan-MGB probe were designed targeting the internal transcribed spacer 2 (ITS2) region of *Dictyocaulus filaria*. The assay showed a strong specificity for detecting *Dictyocaulus filaria*, and had no cross-reactivity with other control pathogenic parasites. It also exhibited high sensitivity, with both the lower limit of quantification (LLOQ) and the limit of detection (LOD) determined to be 1.5 copies per reaction. The assay exhibited excellent repeatability and reproducibility, with an intra-assay coefficient of variation (CV) of 0.11–0.58% and an inter-assay CV of 0.33–2.19%. Finally, the developed TaqMan-MGB probe-based qPCR was used to detect 200 clinical samples. The results indicated that the positive detection rate of *Dictyocaulus filaria* was 3.5% (7/200) for *Dictyocaulus filaria*, and this finding showed good consistency with conventional PCR. The developed TaqMan-MGB probe-based qPCR assay offers several advantages, including high sensitivity, strong specificity, high throughput, speed, convenience, and cost-effectiveness. It is of great significance for safeguarding animal quality and protecting human health.

## Introduction

Lungworm disease caused by *Dictyocaulus filaria* (Rudolphi, 1809) is an infectious condition affecting sheep and goats worldwide. It can pose serious threats to animal quality, human health, and overall public health security (1–3). *Dictyocaulus filaria* resides in the trachea and bronchioles, where the host's response to infection leads to bronchitis, eosinophilic pneumonia, and respiratory bronchiole obstruction. This can cause clinical signs such as dyspnea, coughing, fever, loss of appetite, poor growth and development, and even death (4–7).

Accurate diagnosis of lungworm disease using reliable detection techniques is essential for formulating targeted control measures, which are crucial for the effective management of *Dictyocaulus filaria*.

At present, the diagnosis of *Dictyocaulus filaria* depends on microscopic examination methods, such as the Baermann technique, which is used to identify first-stage larvae in feces (8, 9). However, microscopic examination may easily miss cases with less apparent parasite morphological characteristics, potentially resulting in environmental pollution and endangering the safety of inspectors.

The enzyme-linked immunosorbent assay (ELISA) is suitable for large-scale sample testing, but it involves multiple steps and requires a long experimental duration. It is usually used to detect antibodies in serum. However, in the early stages of infection, the body may not have produced sufficient relevant antibodies or antibody levels may not have reached the detection limit, increasing the likelihood of false negative results. In addition, cross-reactions between *Dictyocaulus filaria* and certain other parasites can easily lead to false positive results, thereby affecting the specificity of the detection method (10).

Therefore, reliable detection techniques are needed for the timely and accurate diagnosis of lungworm disease to prevent its widespread transmission. Due to the challenges associated with serologic testing, molecular testing has become the preferred diagnostic test for detecting certain pathogenic agent infections. In parasitological diagnosis, molecular detection offers high specificity because it can detect the specific nucleic acid of parasites. This approach also effectively addresses the “bottleneck” problem associated with traditional microscopic examination, which often misses early infection cases.

With advancements in molecular biology, polymerase chain reaction (PCR) has been increasingly used for the auxiliary diagnosis of lungworm disease and the detection and identification of *Dictyocaulus* lungworms (11–14). However, conventional PCR has some limitations, such as complex procedures, a high risk of contamination, and challenges in providing accurate and quantitative detection.

Random amplified polymorphic DNA (RAPD)-PCR and PCR/ restriction fragment length polymorphism (RFLP) methods compare banding patterns to assess the degree of genetic differences between different lungworms (Dictyocaulidae). However, these methods require PCR amplification, enzyme digestion identification, and agarose gel electrophoresis to analyze the results (15, 16).

Loop-mediated isothermal amplification (LAMP) offers high amplification efficiency and a short reaction time. However, due to its strong sensitivity, it is particularly prone to aerosol formation, which can lead to false positives and impact the reliability of test results. Therefore, there is an urgent need to develop a rapid, specific, sensitive, and high-throughput detection method for lungworm disease, particularly for early-stage infections of *Dictyocaulus filarial*, as well as for related drugs and biological products.

In recent years, TaqMan probe-based quantitative real-time polymerase chain reaction (qPCR) has been widely used for gene expression analysis, mutation and polymorphism research, and qualitative and quantitative detection of pathogens due to its advantages of high sensitivity, rapid speed, and strong specificity. Compared to conventional TaqMan probe-based qPCR, the minor groove binder (MGB) probe has two key features: First, the 3′ end of the probe is labeled with a non-fluorescent quencher (NFQ). This reduces the fluorescence background and greatly improves the fluorescence spectral resolution. Second, an MGB is attached to the 3′ end of the probe. The MGB can stabilize the hybridization between the probe and the template, increase the annealing temperature of the probe, shorten the length of the probe, and reduce the distance between the fluorophore and the quenching group, thereby enhancing the quenching effect. On the other hand, the specificity of the probe is also improved, resulting in more accurate results and higher resolution (17).

To date, there have been no reports of using TaqMan-MGB probe-based qPCR to detect *Dictyocaulus filaria*. In this study, a specific and sensitive TaqMan-MGB probe-based qPCR method was developed for the first time, which was successfully applied to the rapid detection of *Dictyocaulus filaria* in clinical samples. This provides an effective tool for the molecular diagnosis of lungworm disease.

## Materials and methods

### Design and synthesis of primers and a probe

According to the whole genome sequences of *Dictyocaulus filaria* deposited in GenBank, multiple sequence alignment was performed with the DNASTAR software (version 7.1) to identify the highly conserved internal transcribed spacer 2 (ITS2) region of *Dictyocaulus filaria* as the target gene. Primers and a probe for the ITS2 gene sequences were designed with Primer Express™ Version 3.0 (Thermo Fisher Scientific, Waltham, MA, USA). The specificity of all primers and the probe was checked using the Basic Local Alignment Search Tool (BLAST) provided by the National Center for Biotechnology Information (NCBI) (https://blast.ncbi.nlm.nih.gov/Blast.cgi). No significant similarities were observed. Specific primers and a probe for TaqMan-MGB probe-based qPCR were designed, with the 5′ end labeled with a FAM fluorescence reporter and the 3′ end labeled with an MGB NFQ. The primers and probe were synthesized using Applied Biosystems (ABI, Massachusetts, USA), and their sequences are shown in Table 1. To evaluate the specificity of the primer and probe sets, DNA from *Dictyocaulus filaria* was confirmed by PCR-specific primers serving as templates for amplification using the developed TaqMan-MGB probe-based qPCR. Primers and a probe demonstrating excellent specificity and high sensitivity were identified through experimental screening.

**Table 1:** qPCR primers and TaqMan-MGB probe^#^ sequences used in this study.

**Pathogen**	**Gene**	**Primers/probe**	**Nucleotide sequence**	**Product size**
*Dictyocaulus filaria* (GenBank: OQ110558)	ITS2	GZQDfITS2-F	5′ -GTTTCACGTTACCGTTTTATAATTCATC-3′	70 bp
		GZQDfITS2-R	5′ -ACTGTGCAAATCGTCATCGTTT-3′	
		GZQDfITS2-P	5′ - (FAM)-TTGGCGAGCGGTAAT-(MGB-NFQ)−3′	

^#^All qPCR primers and the TaqMan-MGB probe are protected by patent. For any commercial use please contact the corresponding author.

### Parasites and clinical samples

Clinical samples collected between 2019 and 2023 were preserved at −80°C in our laboratory. These samples were mainly obtained from Beijing and Jilin in China. DNA preserved at −20°C was used as the PCR template. All positive samples were identified by conventional PCR in our laboratory and confirmed by DNA sequencing conducted by Takara Biomedical Technology (Beijing) Co., Ltd.

### DNA extraction

According to the manufacturer's instructions, total DNA was extracted from blood, serum, and anal swab samples using the DNeasy Blood and Tissue Kit (Qiagen, Germany). Parasite DNA was extracted using the QIAamp DNA Mini Kit (Qiagen, Germany).

### Construction of plasmid standards

The target fragments of *Dictyocaulus filaria* were amplified by PCR using the DNA extracted in the previous step. The primers used for amplification were the same as those used in the TaqMan-MGB probe-based qPCR method. The PCR fragments were then cloned into the pMD18-T vector (Takara Biomedical Technology (Beijing) Co., Ltd.) via TA cloning and confirmed by DNA sequencing. Plasmid concentrations were then converted to copy number using the following formula: {plasmid copy number/μL = [6.02 × 10^23^ × plasmid concentration (ng/μL) × 10^−9^] / [plasmid length × 660]}. TaqMan-MGB probe-based qPCR was performed for *Dictyocaulus filaria* using 10-fold diluted plasmids to generate standard curves, based on which, the *E* value (amplification efficiency), *R*^2^ (correlation coefficient), and the standard equation were calculated.

### Optimization of the TaqMan-MGB probe-based qPCR assay

The primer and probe concentrations, along with the annealing temperatures of the TaqMan-MGB probe-based qPCR assay, were optimized to establish the optimal reaction system and conditions for detecting *Dictyocaulus filaria*. Briefly, TaqMan-MGB probe-based qPCR was performed in a 20 μL reaction volume, consisting of 10 μL of TaqMan™ Qiankun Platinum Multiple Premix (A55163, Applied Biosystem, USA), 1 μL of primer/probe mix (0.9 μM of the forward primer GZQDfITS2-F, 0.9 μM of the reverse primer GZQDfITS2-R, and 0.25 μM of the probe GZQDfITS2-P for *Dictyocaulus filaria*), 1 μL of template DNA, and 8 μL of nuclease-free water. Amplification reactions were performed using the QuantStudio™ 6 Real-time PCR System (Applied Biosystems, Massachusetts, USA). The amplification procedure was as follows: 50°C for 2 min (1 cycle), followed by 95°C for 10 min (1 cycle), then 40 cycles of 95°C for 15 s and 60°C for 1 min. Fluorescence signals for each sample were collected at the end of each step at 60°C. Quantification cycle (Cq) values were generated using the QuantStudio Design & Analysis Software with automated threshold settings. Positive and negative controls were included in each assay.

### Specificity of the TaqMan-MGB probe-based qPCR assay

To assess potential cross-reactivity, DNA from other parasites, including *Strongyloides stercoralis, Syphacia obvelata, Syphacia muris*, and *Aspiculuris tetraptera*, was tested to evaluate the specificity of the TaqMan-MGB probe-based qPCR assay using the abovementioned procedure and parameters. *Dictyocaulus filaria* was used as the positive control, while nuclease-free water was used as the negative control.

### The efficiency (*E*), lower limit of quantification (LLOQ), and limit of detection (LOD) of the TaqMan-MGB probe-based qPCR assay

To evaluate the linearity, dynamic range, amplification efficiency (*E*), lower limit of quantification (LLOQ), and limit of detection (LOD) of the TaqMan-MGB probe-based qPCR assay (18–21), serial dilutions of *Dictyocaulus filaria* DNA standards were used as templates. A final standard curve was generated based on the Cq value and the logarithm of the standard copy number. The slope of the linear regression of a plot of Cq (*y*-axis) vs. the log of the target concentration (*x*-axis) was determined by the amplification efficiency (*E*), which was calculated as %*E* = 100 × (−1 + 10 ^(−1/*slope*)^).

### Repeatability and reproducibility of the TaqMan-MGB probe-based qPCR assay

To assess the repeatability and reproducibility of the TaqMan-MGB probe-based qPCR assay, five 10-fold gradient dilutions (1.5 × 10^7^-1.5 × 10^3^ copies/μL) of the DNA standard were tested in triplicate on the same day to evaluate intra-assay repeatability. For inter-assay reproducibility, each dilution was tested in triplicate across three different days within 1 week. The coefficient of variation (CV) for intra- and inter-assay precision was calculated using the following formula: %CV = 100 × [standard deviation (SD) value / mean value].

### Clinical performance of the TaqMan-MGB probe-based qPCR assay

DNA from clinical specimens was tested using the TaqMan-MGB probe-based qPCR assay. When detecting clinical specimens, positive controls (*Dictyocaulus filaria DNA*) and nuclease-free water were included in each run to monitor for false negative and false positive results, respectively.

## Statistical analysis

Statistical analyses for the standard curves were carried out using SPSS Statistics (version: 21.0). Correlation coefficients (*R*^2^) and amplification efficiencies (*E*) of the standard curves were calculated, and the TaqMan-MGB probe-based qPCR amplification curves were visualized using the QuantStudio Design & Analysis Software (version: 1.4.1).

## Results

### Specificity of the TaqMan-MGB probe-based qPCR assay

**Figure 1:**
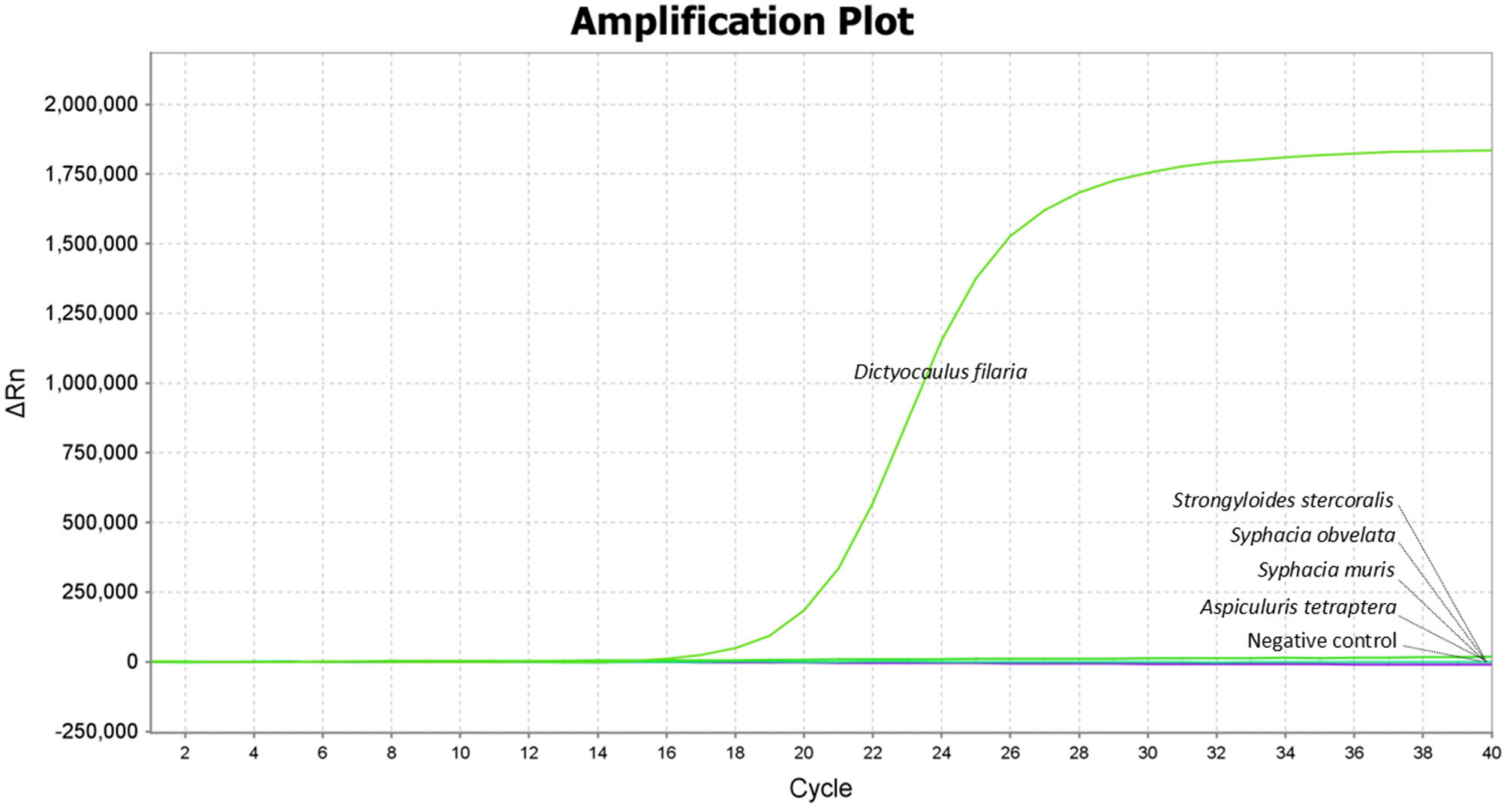
Specificity of the TaqMan-MGB probe-based qPCR assay for detecting *Dictyocaulus filaria*. Amplification curves show the samples positive for *Dictyocaulus filaria* as detected by the TaqMan-MGB probe-based qPCR assay. The negative samples include *Strongyloides stercoralis, Syphacia obvelata, Syphacia muris*, and *Aspiculuris tetraptera*, and nuclease-free water was used as the negative control.

The results showed that only *Dictyocaulus filaria* produced positive fluorescent signals and specific amplification curves, while *Strongyloides stercoralis, Syphacia obvelata, Syphacia muris*, and *Aspiculuris tetraptera* did not demonstrate any positive fluorescent signals or amplification curves, indicating the high specificity of the TaqMan-MGB probe-based qPCR assay (Figure 1).

### Efficiency (*E*) of the TaqMan-MGB probe-based qPCR assay

**Figure 2:**
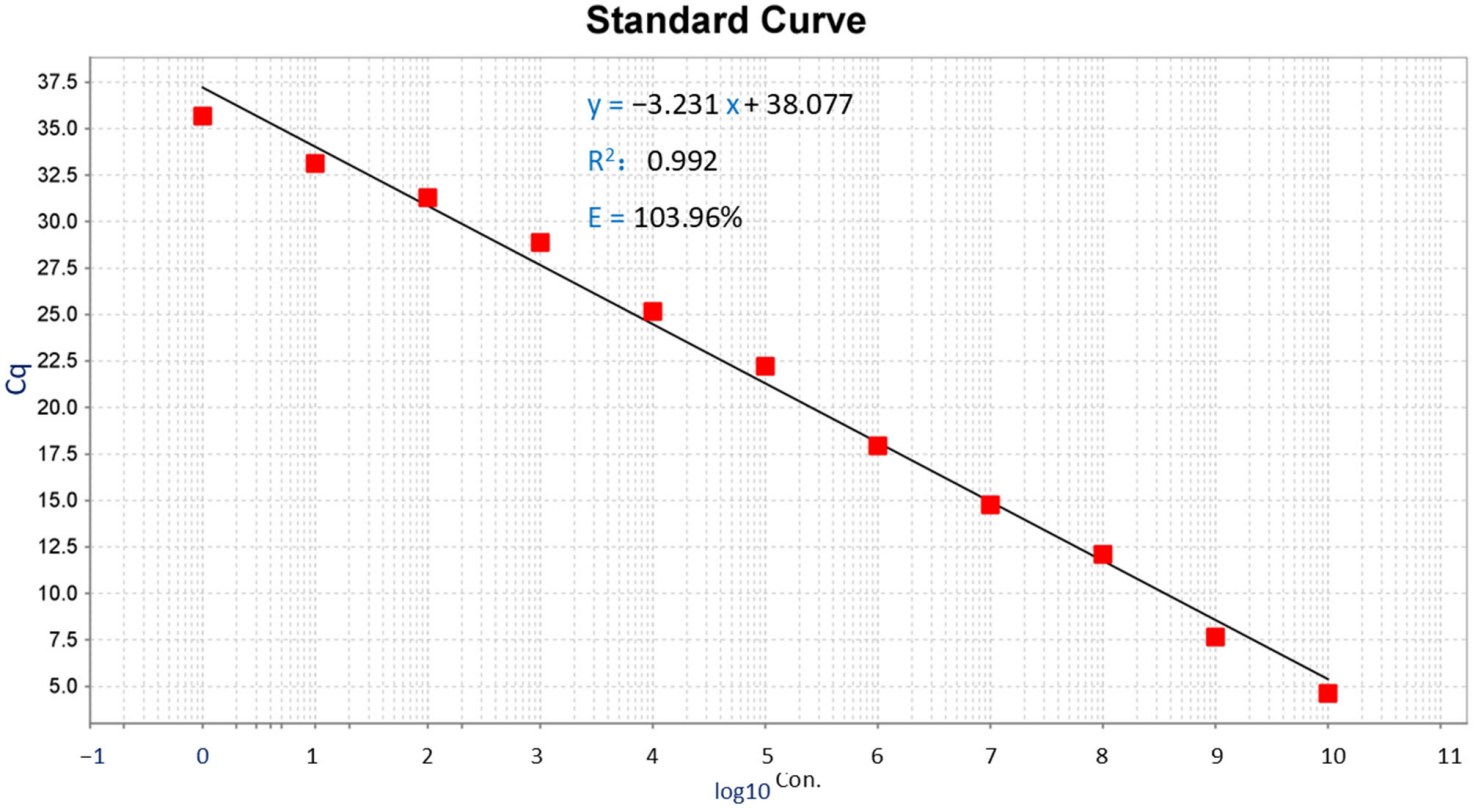
Standard curve of TaqMan-MGB probe-based qPCR for detecting *Dictyocaulus filaria*. Standard curves of *Dictyocaulus filaria* for plasmid standards at concentrations ranging from 1.5 × 101° to 1.5 × 10° copies per reaction.

To construct a standard curve with the logarithm of the DNA copy number and the measured Cq values, serial 10-fold gradient dilutions of the DNA standards at concentrations ranging from 1.5 × 10^10^ to 1.5 × 10^0^ copies/μL were tested. Three replicates were tested for each dilution in a single reaction. The optimal curves were selected as the standard curves (Figure 2).

Establishment of the *Dictyocaulus filaria* standard curve: The abscissa represents the logarithm of the copy number, and the ordinate represents the Cq value. The correlation coefficient was 0.992, the slope of the equation was −3.231, the intercept was 38.077, and the amplification efficiency (*E*) of *Dictyocaulus filaria* was 103.926%. The standard formula for *Dictyocaulus filaria* was *y* = −3.231*x* + 38.077 (Figure 2, the y-axis represents the Cq value, and the *x-*axis represents the logarithm of the concentration). The correlation coefficient of the standard curves for *Dictyocaulus filaria* was 0.992, suggesting that the correlation between each quantified concentration group of the standards was reliable. The amplification efficiency of *Dictyocaulus filaria* was 103.926%, which is considered satisfactory for TaqMan-MGB probe-based qPCR.

### Lower limit of quantification (LLOQ), and limit of detection (LOD) of the TaqMan-MGB probe-based qPCR assay

**Figure 3:**
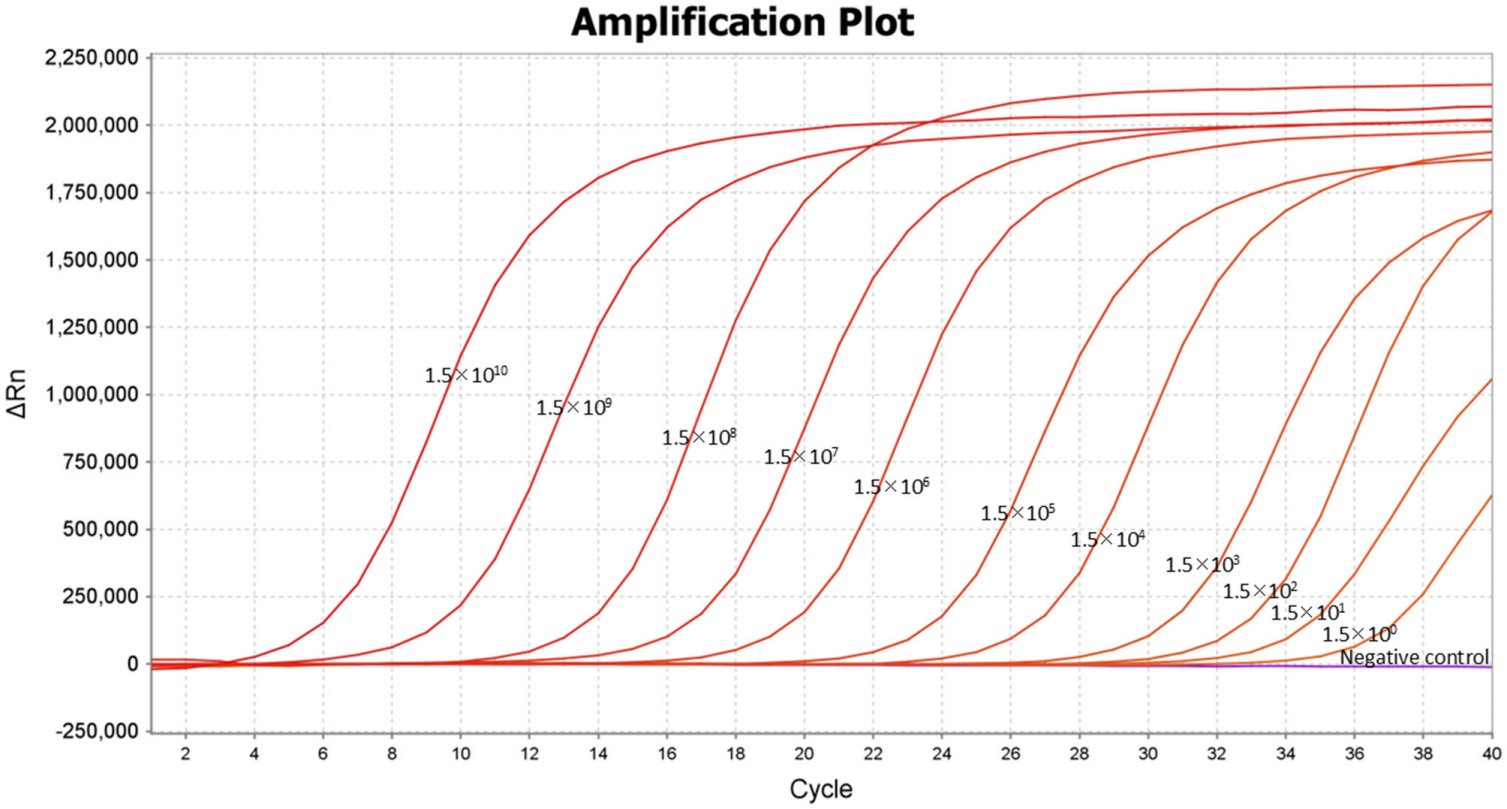
Lower limit of quantification (LLOQ) of the TaqMan-MGB probe-based qPCR assay for detecting *Dictyocaulus filaria*. Amplification curves of *Dictyocaulus filaria* obtained using serial 10-fold dilutions of the DNA standards, ranging from 1.5 × 10^10^ copies/μL to 1.5 × 10^0^ copies/μL.

The 10-fold gradient dilutions of the DNA standard were tested using the developed TaqMan-MGB probe-based qPCR. The lower limit of quantification (LLOQ) of the method was 1.5 × 10^0^ copies/μL for detecting *Dictyocaulus filaria* (Figure 3), indicating that the TaqMan-MGB probe-based qPCR was highly sensitivity.

The two-fold gradient dilution of the DNA standard was tested using the developed TaqMan-MGB probe-based qPCR. The limit of detection (LOD) for this method was defined as the lowest concentration of the target analyte, which can be detected with a 95% detection rate. Follow-up experiments showed that the detection rate of the samples at 1.5 copies/μL for detecting *Dictyocaulus filaria* was 100% across 20 replicates (Table 2). Thus, the reliable LOD of the TaqMan-MGB probe-based qPCR assay was 1.5 copies per reaction for detecting *Dictyocaulus filaria*. In this experiment, the cutoff line for positivity was automatically determined by the QuantStudio™ 6 Real-time PCR Instrument. We set the cutoff line for positivity at 38, meaning that the samples with a Cq value less than or equal to 35 (≤35) were regarded as positive, those with Cq values greater than 35 but less than or equal to 38 (35 < and ≤ 38) were considered invalid, and those with Cq values greater than 38 (>38) were considered negative. The criteria were established based on two considerations. First, the LOD of the method was 1.5 copies/μL, with a corresponding Cq value of ~35. Second, although some samples at 1.5 copies/μL were detectable, their Cq values were ~35.71. All subsequent experiments were conducted in accordance with these criteria. Briefly, if there was a specific S-shaped curve and the Cq value was ≤ 35, the result was determined to be positive for *Dictyocaulus filaria* nucleic acid. If there was no Cq value and no specific fluorescence amplification curve, the result was interpreted as negative for *Dictyocaulus filaria* nucleic acid. If the Cq value was between 35 and 38 (35 < Cq value < 38) and there was a specific fluorescence amplification curve, the result was interpreted as suspicious for *Dictyocaulus filaria* nucleic acid, and the suspicious samples were resampled for DNA extraction and retesting. If the Cq value was < 38, the result was interpreted as positive; otherwise, the result was considered negative.

**Table 2:** Limit of detection (LOD) of the TaqMan-MGB probe-based qPCR assay for detecting *Dictyocaulus filaria*.

**Pathogen**	**Concentration**	**Repeat times**	**Positive number**	**Positive rate**	**95% positive rate**
*Dictyocaulus filaria*	15 copies/μL	20	20	100%	>95%
	7.5 copies/μL	20	20	100%	>95%
	3.75 copies/μL	20	20	100%	>95%
	1.5 copies/μL	20	20	100%	>95%

The cutoff line for positivity was automatically determined by the QuantStudio™ 6 Real-time PCR system.

### Repeatability and reproducibility of the TaqMan-MGB probe-based qPCR assay

As shown in Table 3, the TaqMan-MGB probe-based qPCR assay exhibited intra-assay CVs ranging from 0.11% to 0.58% for *Dictyocaulus filaria*. The TaqMan-MGB probe-based qPCR assay showed inter-assay CVs ranging from 0.33% to 2.19% for *Dictyocaulus filaria*. The results showed that both intra- and inter-assay CVs were below 3%, indicating good repeatability (intra-assay) and reproducibility (inter-assay) of the TaqMan-MGB probe-based qPCR assay.

**Table 3:** Repeatability and reproducibility of the TaqMan-MGB probe-based qPCR assay for detecting *Dictyocaulus filaria*.

**Item**	**Concentration**	**Cq value 1**	**Cq value 2**	**Cq value 3**	**Cq mean ±SD**	**CV**
Intra-assay	1.5 × 10^7^ copies/μL	15.57	15.64	15.77	15.66 ± 0.09	0.57%
	1.5 × 10^6^ copies/μL	18.76	18.81	18.98	18.85 ± 0.11	0.58%
	1.5 × 10^5^ copies/μL	22.93	23.08	23.10	23.03 ± 0.09	0.39%
	1.5 × 10^4^ copies/μL	25.97	26.01	26.04	26.00 ± 0.03	0.11%
	1.5 × 10^3^ copies/μL	29.73	29.75	29.89	29.79 ± 0.08	0.26%
Inter-assay	1.5 × 10^7^ copies/μL	15.83	16.34	15.66	15.94 ± 0.35	2.19%
	1.5 × 10^6^ copies/μL	18.81	18.63	18.89	18.77 ± 0.13	0.69%
	1.5 × 10^5^ copies/μL	22.99	22.70	22.65	22.78 ± 0.18	0.79%
	1.5 × 10^4^ copies/μL	25.94	26.31	26.61	26.28 ± 0.33	1.25%
	1.5 × 10^3^ copies/μL	29.76	29.75	29.93	29.81 ± 0.10	0.33%

### Clinical performance of the TaqMan-MGB probe-based qPCR assay

**Figure 4:**
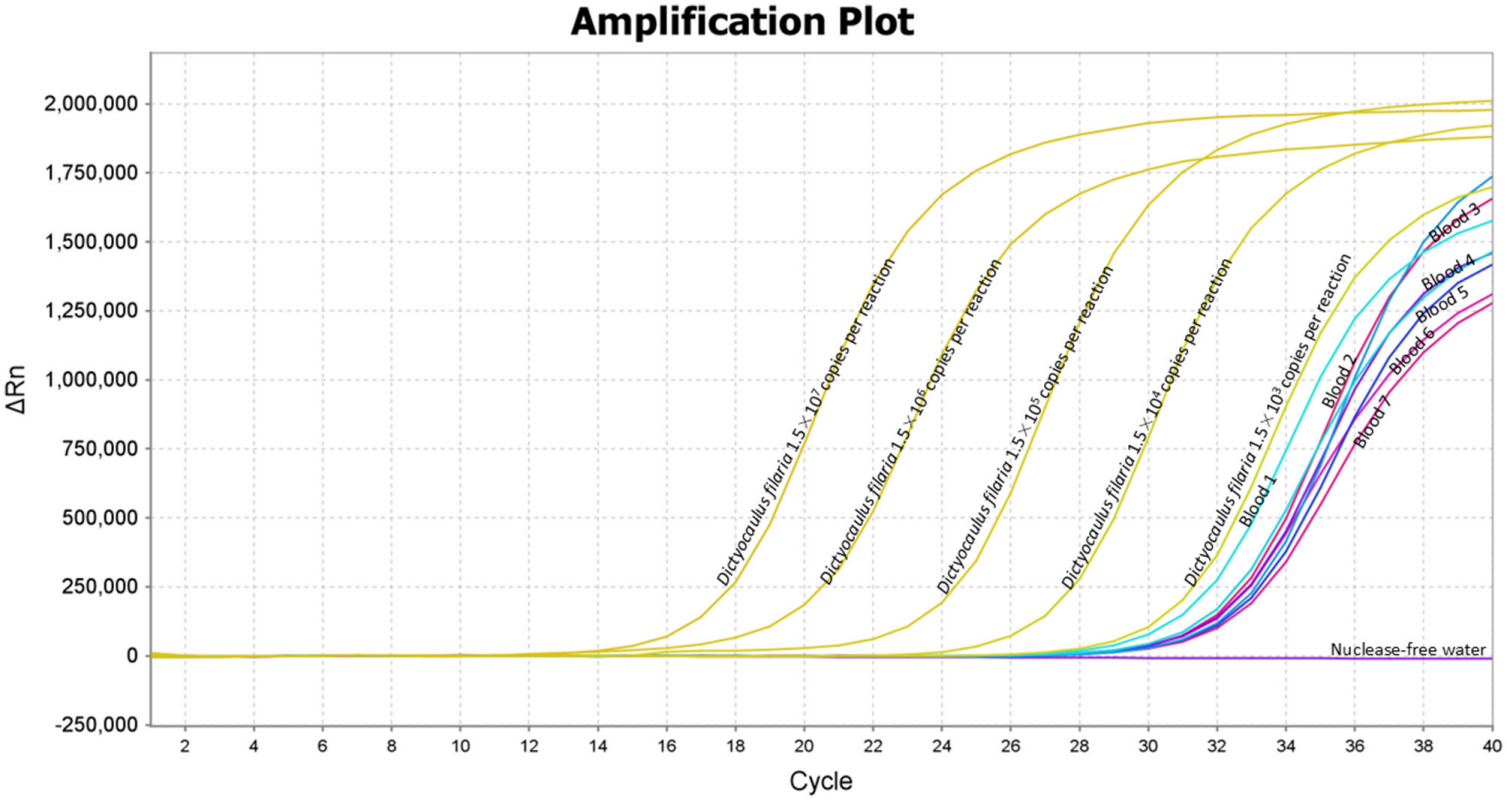
Application of the TaqMan-MGB probe-based qPCR assay for detecting *Dictyocaulus filaria* in the clinical samples. The clinical samples revealed positive results for *Dictyocaulus filaria* in the seven blood samples, indicated by positive fluorescent signals, specific amplification curves, and quantification cycle (Cq) values ≤ 35. Amplification curves of *Dictyocaulus filaria* for plasmid standards at concentrations ranging from 1.5 × 10^7^ to 1.5 × 10^3^ copies per reaction.

**Figure 5:**
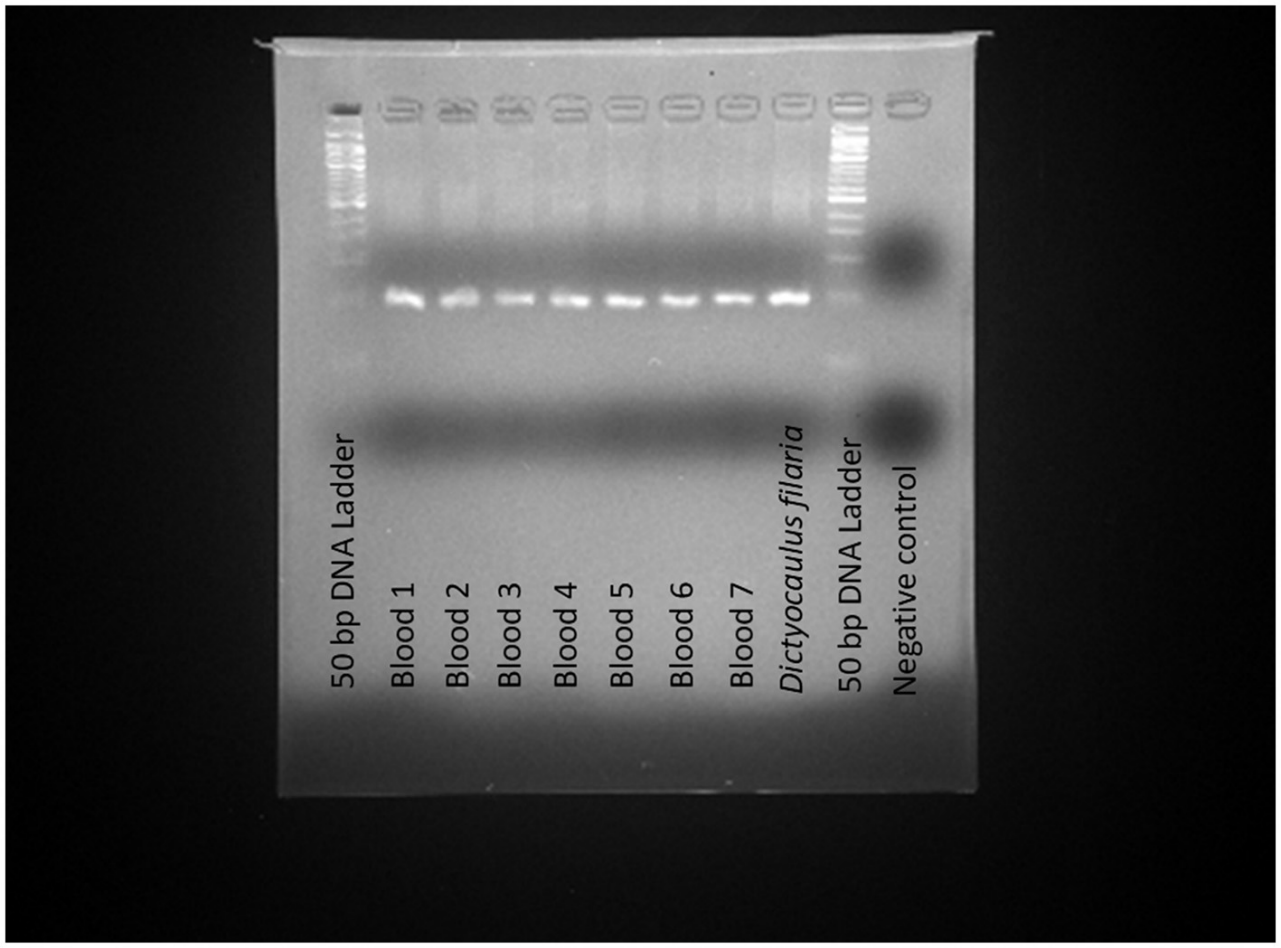
Application of conventional PCR for the detection of *Dictyocaulus filaria* in the clinical samples. The clinical samples revealed positive results for *Dictyocaulus filaria* in the seven blood samples, indicated by the presence of a positive 103 bp fragment on agarose gel electrophoresis. Positive control: *Dictyocaulus filaria*; Negative control: nuclease-free water.

The application of the TaqMan-MGB probe-based qPCR assay to clinical samples detected *Dictyocaulus filaria* in seven blood samples, evidenced by positive fluorescent signals, specific amplification curves, and cycle threshold values ≤ 35 (Figure 4). The TaqMan-MGB probe-based qPCR assay showed that 3.5% (7/200) of the samples were positive for *Dictyocaulus filariai*. Notably, all seven samples also tested positive for *Dictyocaulus filaria* when confirmed using conventional PCR and sequencing methods (Figure 5). A brief, the total volume of the conventional PCR reaction was 50 μL, consisting of 5 μL of 10 × Buffer (Mg^2+^ Plus), 4 μL of dNTP mixture, 0.5 μL of the forward primer GZQDfITS2-CF (10 μM), 0.5 μL of the reverse primer GZQDfITS2-CR (10 μM), 0.25 μL of EX Taq (5 U/μL) [Takara Biomedical Technology (Beijing) Co., Ltd.], 2 μL of template DNA, and 37.75 μL of nuclease-free water. Amplification was carried out on the Veriti 96-Well Thermal Cycler equipment (Applied Biosystems) using the following program: 94°C for 5 min, 1 cycle; 95°C for 30 s, 60°C for 30 s, and 72°C for 1 min, 35 cycles; and 72°C for 10 min, 1 cycle. The PCR amplification products were detected using 3% agarose gel electrophoresis. The positive PCR amplification products were confirmed using the sequencing method. The conventional PCR primers were synthesized by Takara Biomedical Technology (Beijing) Co., Ltd. and are shown in Table 4.

**Table 4:** Conventional PCR primer sequences used in this study.

**Pathogen**	**Gene**	**Primers/probe**	**Nucleotide sequence**	**Product size**
*Dictyocaulus filaria* (GenBank: OQ110558)	ITS2	GZQDfITS2-CF	5′ -GCTGCGAGTTTCACGTTACC-3	103 bp
		GZQDfITS2-CR	5′ -ATCTGCACACAACACGCTCT-3′	

## Discussion

*Dictyocaulus filaria* infection remains a major financial concern due to its impact on both production losses and animal health and welfare (22). There is no effective vaccine available for the prevention and control of this disease (23–25). The control of *Dictyocaulus filaria* infestation in sheep relies on pasture management, the development of host immunity against the parasite, and the prudent use of a suitable anthelmintic (26–28). Indeed, in the context of increasing levels of resistance to anthelmintics (29) and growing concerns regarding the environmental impact of anthelmintic drug residues (30, 31), it is important to implement a safe strategy for parasitism management at the farm level that includes the accurate use of detection tools.

In view of this situation, an effective way to prevent the disease is to detect and eliminate positive individuals step by step. In this study, a novel TaqMan-MGB probe-based qPCR for detecting *Dictyocaulus filaria* was developed for the first time.

Specific primers and a probe targeting the conserved region of ITS2 were selected as detection molecular markers. The ITS2 region is moderately conserved, and its conservation is basically manifested as relative consistency within parasite species and distinct differences between different parasite species. This characteristic makes ITS2 suitable for the molecular identification of *Dictyocaulus filaria*. The method successfully detected *Dictyocaulus filaria* without any cross-reaction with other parasites, indicating the high specificity and reliability of this method for *Dictyocaulus filaria* detection.

For *Dictyocaulus filaria DNA*, the minimum detection limit of the developed TaqMan-MGB probe-based qPCR method was 1.5 copies per reaction. Accurate detection of *Dictyocaulus filaria* at lower concentrations enables early diagnosis and prevention of lungworm disease. TaqMan-MGB probe-based qPCR provides improved monitoring of *Dictyocaulus filaria* and is expected to enhance biosafety in sheep farm management.

However, the improvement in sensitivity also increases the risk of false positive results, which demands stricter contamination prevention during sampling and analysis. Good laboratory practices are necessary to obtain reliable results. An excellent and efficient detection method should accurately reflect epidemiological data—such as disease prevalence, transmission rate, and epidemic severity—thereby facilitating effective monitoring and prevention of the disease.

The developed TaqMan-MGB probe-based qPCR assay offers improved detection capabilities in a shorter time and at a lower labor cost. However, due to technical difficulties encountered during development, establishing this method remains more challenging compared to conventional PCR To the best of our knowledge, this is the first TaqMan-MGB probe-based qPCR detection method developed specifically to detect *Dictyocaulus filaria*, a parasite commonly found in sheep. This method can ensure good specificity and sensitivity and will undoubtedly save labor and material costs. The established detection method can detect *Dictyocaulus filaria* in clinical samples, making the detection process more convenient.

## Conclusion

In this study, we designed specific primes and a probe based on the conserved region of the *Dictyocaulus filaria* sequence, constructed plasmid standards and standard curves, and developed a novel TaqMan-MGB probe-based qPCR detection method characterized by simple operation, high sensitivity, strong specificity, good stability, and a wide dynamic range. This assay can detect *Dictyocaulus filaria* qualitatively, quantitatively, rapidly, and accurately in different clinical samples. This is of great significance for safeguarding animal quality and human health. This assay has significant practical value in the quality control of animal-derived biological products intended for human use, as well as in the inspection and quarantine of animals for import and export. It deserves broad application in these fields.

## Data Availability

The datasets presented in this study can be found in online repositories. The names of the repository/repositories and accession number(s) can be found in the article/supplementary material.
